# Circulating tumor cells as a preoperative risk marker for occult metastases in patients with resectable cholangiocarcinoma

**DOI:** 10.3389/fonc.2022.941660

**Published:** 2022-11-10

**Authors:** Thorben Fründt, Johann von Felden, Jenny Krause, Asmus Heumann, Jun Li, Sabine Riethdorf, Klaus Pantel, Samuel Huber, Ansgar W. Lohse, Henning Wege, Kornelius Schulze

**Affiliations:** ^1^ Department of Medicine, University Medical Center Hamburg-Eppendorf, Hamburg, Germany; ^2^ Department of General, Visceral and Thorax Surgery, University Medical Center Hamburg-Eppendorf, Hamburg, Germany; ^3^ Department of General Surgery, Surgical Oncology, Jiahui International Hospital, Shanghai, China; ^4^ Institute of Tumorbiology, University Medical Center Hamburg- Eppendorf, Hamburg, Germany; ^5^ Department of Internal Medicine, Cancer Center Esslingen, Oncology/Hematology, Gastroenterology and Infectious Diseases, Esslingen, Germany

**Keywords:** cholangiocarcinoma, circulating tumor cells, liquid biopsy, occult metastases, cellsearch system

## Abstract

Cholangiocarcinoma (CCA) is an aggressive tumor associated with a high rate of recurrence after resection. An important risk factor for recurrence is the presence of occult metasta-ses, which are not radiologically detectable at the time of diagnosis. There are currently no biomarkers for the preoperative assessment of micrometastases. A previous study demonstrated the prognostic relevance of circulating tumor cells (CTC) in patients with advanced CCA but the potential of CTCs as a preoperative marker for detecting occult metastases has not been investigated so far. In this two-phase study, we first recruited a cohort of 27 patients with histologically proven, metastatic CCA or gallbladder cancer (GBCA) to assess feasibility (feasibility cohort, FC). CTCs were measured in the peripheral blood using the CellSearch System (CSS) between October 2012 and January 2017. Subsequently, in 11 patients undergoing curative-intended resection for CCA (intrahepatic CCA: n =4; extrahepatic CCA n= 6; gallbladder cancer: n=1), peripheral and central venous blood specimens were obtained to improve detection rate by simultaneous measurement and to elucidate distribution of CTCs in different venous compartments. Presence of CTCs detection was correlated with postoperative TNM-status.

In the FC, CTCs (range 1-3 cells, median: 1) were detected in 40% (11/27) patients and were signifi-cantly associated with worse overall survival (hazard ratio: 3.59; 95% CI: 1.79- 7.1; p = 0.04). By combined peripheral and central measurement, CTC detection was increased to 54% (6/11) in the resection cohort (RC) and was associated with metastases that were only identified during the surgical procedure (peritoneal carcinoma: n = 1; infiltration of the duodenum: n = 1) or immediately after surgery (evidence of pulmonary metastases by CT scan two days after resection, not evident on initial tumor staging prior resection). Taken together, in this single center pilot study, we demonstrated that CTCs are detectable in CCA patients and are associated with significantly impaired survival in patients at metastatic stage. Detection rate prior to surgery was improved to >50% by combined peripheral and central measurement. Moreover, preoperative CTC detection may indicate existing metastases and could help to stratify patients more accurately.

## Introduction

Cholangiocarcinoma (CCA) is the second most primary liver tumor, accounting for approximately 15% of all liver cancers worldwide and for 3% of all gastrointestinal malignancies (1–3). The tumor, arising at any part of the bile duct tree, is categorized by its anatomical location in intrahepatic (iCCA), perihiliar (pCCA) or distal CCA (dCCA). Insertion of the cystic duct presents the margin to separate pCCA from dCCA (4). With approximately 50-60% of all cases, pCCA is the most prevalent subtype of CCA and is sometimes grouped together with dCCA as extrahepatic CCA (eCCA) (5–7).

Several risk factors for tumor development have been identified such as primary sclerosing cholangitis (PSC), fluke infection, chronic hepatitis B (HBV) or hepatitis C (HCV) infection or choledochal cyst, par-tially varying in the association for iCCA or eCCA development and thus leading to a worldwide disparity in tumor incidence (8–10). According to this, an increasing incidence of iCCA has been reported in recent studies. Furthermore, CCA related mortality has also increased within the past year, especially in high-prevalence regions in Asia, underlining the important disease burden caused by CCA (11–13).

Primary tumor resection remains the only curative treatment, but only 26% of patients are eligible for surgery at initial diagnosis. Unfortunately, even following resection, the 5-year survival rate remains poor due to tumor relapse, primarily due to occult metastases that have already spread at the time of surgery (13–16). In patients undergoing curative resection, lymph node involvement, resection margin status, and tumor differentiation have been identified as independent prognostic risk factors for dis-ease free survival. However, as these factors can only be assessed post-surgery, biomarkers allowing a preoperative risk stratification for micrometastases are urgently needed (17, 18).

Circulating tumor cells (CTC) are shed into the bloodstream from a primary tumor or distant metastases and are regarded as metastatic precursors, enabling tumor spread (19, 20). CTCs have a short livespan, are found in scare concentrations sometimes even only one cell per five million white blood cells, and may be detected by a variety of analytic systems using different techniques for cell identification (21). Until today, only the CellSearchTM systems (CSS) has been cleared by the US Food and Drug Administra-tion (FDA) for CTC measurement in carcinoma patients. Prognostic relevance of CTCs has been initially reported for patients with breast cancer by Cristofanilli et al. in 2004, and since then has been demon-strated for several tumors like colorectal, pancreas, or small lung cell cancer, for example (22–26). In terms of liver tumors, prognostic impact of CTCs has been demonstrated for hepatocellular carcinoma (HCC) regarding risk of recurrence following tumor resection and for overall survival (OS) in patients receiving palliative treatment (27–29). In 2012, Al Ustwani et al. first demonstrated that CTCs are de-tectable in patients with CCA and gallbladder cancer (GBCA) (30). Finally, in 2016, Yang et al. described the prognostic relevance of CTC in CCA patients in terms of OS. However, until now, little is known about the ability of CTC measurement to detect occult metastases prior to curative intended surgery (31). Furthermore, it is still unclear if distribution of CTC differs in CCA patients between the peripheral and central venous compartment, as it seems more likely to find a higher amount of CTCs in vessels that are closer located to tumor, regarding the short livespan of CTCs, dilution and trapping of cells in the lung. Therefore, we conducted a prospective study, evaluating the prognostic value of CTC in a single center training cohort. Based on these investigations, we assessed the preoperative prevalence of CTC, their correlation to tumor characteristics, and CTC distribution in different vascular compartments in patients undergoing curative-intended surgery.

## Materials and methods

### Patients

This study was conducted in a two-phase approach: In the initial phase, CTC were measured in peripheral venous blood samples of patients with histologically confirmed, progressed CCA or GBCA, to assess the feasibility of the CSS (feasibility cohort, FC). All patients in this cohort received either palliative systemic treatment or best supportive care, when tumor stage or patients’ performance status rendered systemic chemotherapy impossible. In patients receiving palliative chemotherapy, blood samples were obtained before therapeutic agents were administered. Patients were recruited at the I. Department of Medicine at the University Medical Center Hamburg-Eppendorf between October 2011 und October 2017 and were included in the study, if they were 18 years or older, had a histologically confirmed CCA or tumor of the gallbladder, had no evidence of other solid organ tumors, and gave written informed consent.

In a second phase, CCA patients undergoing curative tumor resection were enrolled between October 2017 and April 2018 (resection cohort, RC). Blood specimens for CTC analyses were obtained immediately prior to surgery from a peripheral cubital vein and from the vena cava superior *via* central vein catheter, that had been placed at the day of surgery. Patients, in whom diagnosis of CCA or gallbladder tumor was not confirmed following resection or who had a history of concurrent solid organ tumors were excluded from further analysis. Following resection, all patients were seen at the outpatient clinic of the I. Department of Medicine, University Medical Center of Hamburg Eppendorf, on regularly intervals. Contrast enhanced CT-scans of the thorax and abdomen were performed every three months for tumor surveillance. Diagnosis of tumor recurrence was confirmed by an interdisciplinary tumor conference.

Follow-up for both cohorts was completed in December 2019. Electronic patient files were reviewed to assess the clinical course, baseline demographic and tumor characteristics. The period of follow-up was determined based on the date when blood specimens were obtained until patient’s death or most cur-rent follow-up visit.

### Blood specimens

Blood samples were collected following puncture of a cubital vein (training and resection cohort) or *via* central vein catheter (resection cohort only). A total of 7.5 mL whole blood was collected using a CellSave Preservative Tube (Veridex). Tubes were stored at room temperature and processed within 96 hours after collection. In order to avoid contamination by epithelial cells from the skin, an additional tube was filled prior to the sample tube.

### CTC analysis

**Figure 1:**
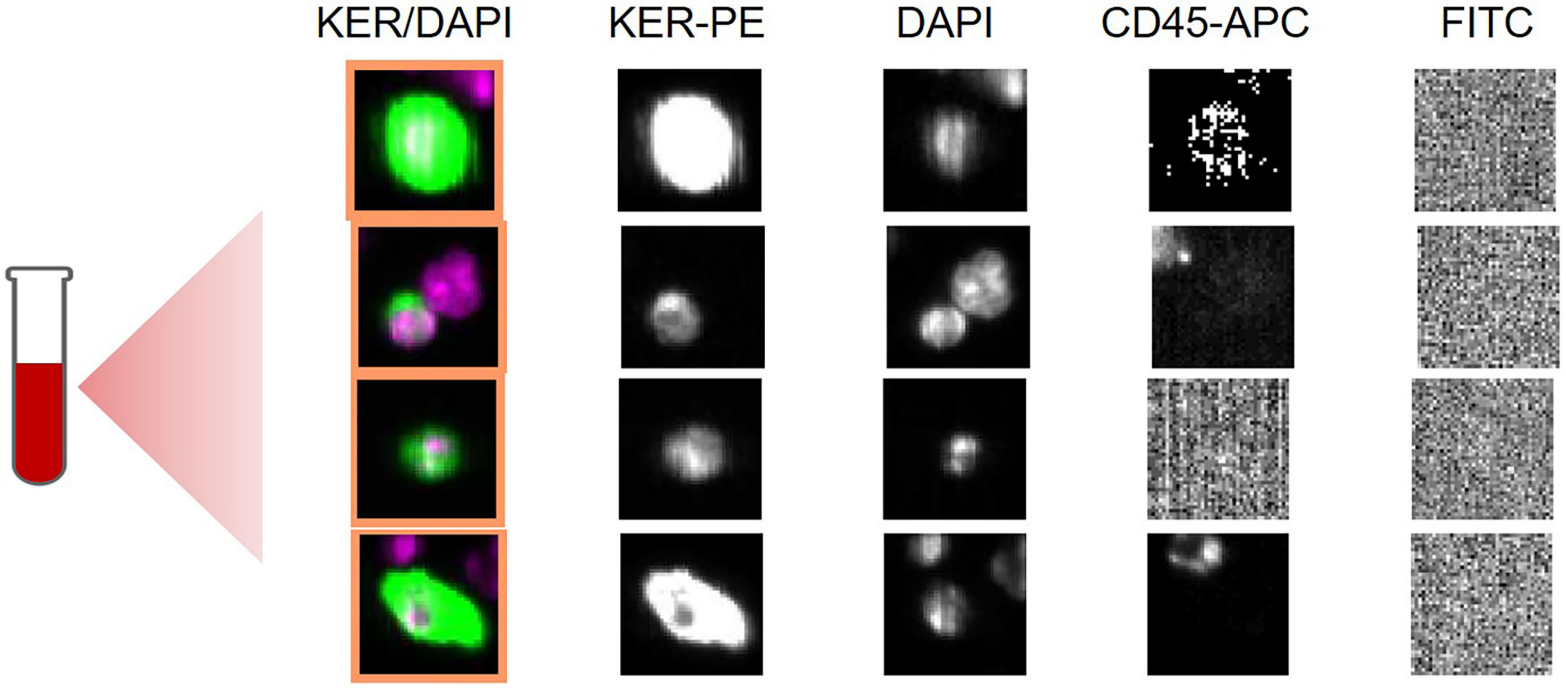
Selected CellSearch images showing circulating tumor cells (CTCs) in an exemplary patient of the training cohort. Blood specimen had been obtained from a 72- year old patient with metastatic iCCA, who received palliative chemotherapy. Each row shows a CTC detected in sample of 7,5 ml of whole blood. Nucleated cells (DAPI positive) that are positive for keratin (KER) and negative for CD45 with a diameter of at least 4 µm are called CTCs. KER/DAPI represent composite images. PE: phycoerythrin; APC: allophycocyanine, FITC: Fluorescein isothiocyanate, DAPI: (4′,6-diamidino-2-phenylindole).

The CSS is a semiautomated device providing detection of CTCs harboring the epithelial cell adhesion molecule (EpCAM), a transmembrane glycoprotein that is expressed in epithelial tumors and is associ-ated with cancer progression and invasiveness (32, 33). Because a high expression of EpCAM has been found in CCA and GBCA, the CSS, providing an EpCAM-based CTC measurement was used in our study (34). In an initial step, blood specimens are incubated with ferrofluid-coated anti-EpCAM antibodies to capture EpCAM positive cells by using the automated Celltracks AutoPrep system. Subsequently, cells are immunostained with fluorescent-labeled anti-keratin antibodies to identify keratins (KER), e.g., 8, 18, and 19. A nuclear staining with 4,6-diamidino-2-phenylindole (DAPI) is added to ensure integrity of nuclei and an anti-CD45 antibody is used to distinguish epithelial cells from leukocytes (**Figure 1**). Following immunostaining, EpCAM positive cells are identified and quantified by the CellTracks Analyzer, a semiautomated fluorescence-based microscopy, with generation of images. Images are analyzed by a blinded and experienced observer on a computer desktop. Two observers independently evaluate computer-proposed images for CTCs excluding leukocytes and artifacts. Evidence of ≥ 1 CTC per 7.5 ml blood sample was defined as positive CTC detection.

### Ethical approval

Blood collection and all experiments were performed in compliance with the Helsinki Declaration and were approved by the local ethics committee, the Ethik-Kommission der Ärztekammer Hamburg, Ham-burg, PV-3578.

### Statistical analysis

Patient demographic data, tumor characteristics, and clinical course were obtained by reviewing digital medical files of all included patients. Categorical variables were described in terms of percentages and frequencies; continuous variables were described in terms of median with minimum-to-maximum range. Median overall survival (mOS) was compared using Kaplan–Meier curves with the log-rank test. Fisher’s exact test was used to compare distribution of count data between groups. Kruskal-Wallis rank sum test was used to compare continuous variable. Chi-Square test was used to compare categorical variables. For all outcomes received, a p value <0.05 indicated statistical significance. All statistical analyses were conducted using Graph Pad Prism 8.4.3 (GraphPad Software ^®^) and SPSS statistic soft-ware Version 26.0 (IBM ^®^).

## Results

CTC analyses were performed in 27 patients with metastatic CCA or GBCA (FC) and 11 patients undergoing curative-intended surgery (RC). Demographic and tumor characteristics are depicted in **Table 1** (FC) and **Table 2** (RC), CTC count and laboratory values for FC patients are shown in the supplements (**Supplement Table 1**). In the FC, 66% (n=18) were male, median age was 72 years (range: 27- 89), 26% (n = 7) had iCCA, pCCA was found in 55% (n = 15) dCCA in 7% (n = 2) and 11% (n = 3) had GBCA. CTC were detected in 41% (n = 11) patients, mean CTC count was 1 (range: 1-3), in three patients (27%) ≥ 2 CTC were found. CTC were predominantly found in patients with pCCA (n = 6) and GBCA (n = 3). Palliative treatment of FC patients included chemotherapy (n = 7; gemcitabin and cisplatin: n = 3; erlotinib and bevacizumab: n = 2, ramucirumab: n = 1; capecitabine: n = 1), four patients were treated with radiofrequency ablation or photo-dynamic therapy, sixteen patients received no therapy or were treated with best supportive care.

**Table 1:** Baseline characteristics of the feasibility cohort.

Characteristics	Total	CTC -	CTC +	*p*
***n***	27	16	11	
**Age (median; min/max)**	72 (27– 89)	73 (47- 89)	64 (27- 82)	.12
**Male (n; %)**	18 (66)	9 (75)	9 (82)	.23
**ECOG (n; %):**				
0	6 (22)	4 (25)	2 (18)	1.0
1	7 (26)	3 (19)	4 (36)	.39
2	9 (33)	6 (37)	3 (27)	.38
3	5 (19)	3 (19)	2 (18)	1.0
**Tumor type (n; %):**				
iCCA	7 (26.9)	4 (25)	3 (27.3)	1.0
pCCA	15 (55.5)	10 (62.5)	5 (45.4)	.45
dCCA	2 (7.5)	2 (12.5)	–	.49
GBCA	3 (11.1)	–	3 (27.3)	.056
**Treatment modalities:** Systemic chemotherapy	11	5	6	
BSC	16	5	11	.68
**Tumor characteristics (n;%):**				
T1/T2	16 (59)	10 (62.5)	6 (55)	.71
T3/4	11 (40.1)	6 (37.5)	5 (45)	
N1	8 (29.6)	4 (25)	4 (36.4)	.67
M1	9 (33.3)	3 (18.8)	6 (54.5)	.09
CTC ≥ 2		–	5 (45)	
**Laboratory findings (median/min- max):**				
yGT [U/L]	386 (54- 2308)	403 (54- 2308)	369 (128- 1484)	.84
AP [U/IL]	352 (63- 876)	269 (63- 806)	502 (100- 876)	.12
ASAT [U/L]	66 (17- 173)	54 (17- 172)	72 (34- 172)	.21
ALAT [U/L]	44 (10- 227)	31 (10- 213)	78 (20- 227)	.48
Bilirubin [mg/dl]	1.9 (0.2- 25.8)	1.0 (0.2- 14.7)	3.5 (0.2- 25.8)	.16
CA 19-9 [kU/L]	324 (1.3- 45093)	394 (9.6- 45093)	152.4 (1.3- 4393.4)	.34

ALT, alanine aminotransferase; AP, alkaline phosphatase; AST, aspartate ami-notransferase; CA 19-9, carbohydrate antigen 19-9; CTC+, CTC positive; CTC-, CTC negative; ECOG, Eastern Cooperative Oncology Group performance index; γGT, gamma-glutamyltransferase.

**Table 2:** Baseline characteristics of the resection cohort.

Characteristics	Total	CTC -	CTC +	*p*
***n***	11	5	6	
**Age (median; min/max)**	67 (50- 79)	67 (60- 73)	65 (50- 79)	.25
**Male (n; %)**	9 (82)	3 (60)	6 (100)	.23
**ECOG (n; %):**				
** 0**	9 (82)	4 (80)	5 (83)	.87
** 1**	2 (18)	1 (20)	1 (17)	
**Tumor type (n; %):**				
** iCCA**	4 (26.9)	2 (40)	2 (33)	.89
** pCCA**	6 (55.5)	2 (40)	4 (66)	
** GBCA**	1 (11.1)	1 (20)	–	
**Tumor characteristics (n;%):**				
** pT1/T2**	7 (64)	2 (40)	5 (83)	.74
** pT3/T4**	4 (36)	3 (60)	1 (17)	
** pN1**	4 (60)	3 (60)	1 (16)	.55
** pM1**	3 (27)	–	3 (50)	.49
** pL1**	3 (27)	2 (40)	1 (16)	.88
** pV1**	3 (27)	2 (40)	1 (16)	.88
**CTC detection:**				
** pv only**		–	2 (33)	
** cv only**		–	2 (33)	
** pv + cv**		–	2 (33)	
**Laboratory findings (median/min- max):**				
** yGT [U/L]**	221 (41- 886)	221 (58- 886)	285 (41- 868)	
** AP [U/IL]**	285 (139- 970)	161 (139- 288)	288 (282- 970)	
** ASAT [U/L]**	408 (29- 610)	387 (238- 427)	481 (29610)	
** ALAT [U/L]**	285 (34- 749)	285 (141- 364)	357 (34- 749)	
** Bilirubin [mg/dl]**	1.9 (0.4- 4.1)	1.2 (0.9- 4.1)	2.3 (0.4- 3.8)	
** CA 19-9 [kU/L]**	1404 (804- 2849)	1848 (846- 2849)	1383 (804- 1962)	

ALT, alanine aminotransferase; AP, alkaline phosphatase; AST, aspartate ami-notransferase; CA 19-9, carbohydrate antigen 19-9; cv, central venous; CTC+, CTC positive; CTC-, CTC negative; ECOG, Eastern Cooperative Oncology Group performance index; pv, peripheral venous; γGT, gamma-glutamyltransferase.

**Figure 2:**
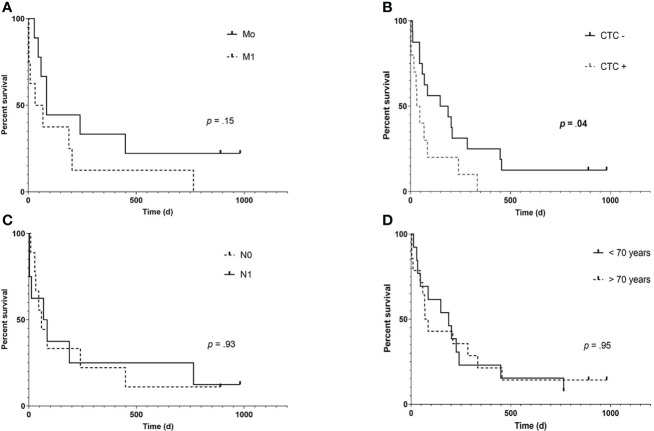
Kaplan- Meier overall survival (OS) estimates according to the presence of distant metastases **(A)**, CTC **(B)**, positive lymph node status **(C)** and older age **(D)** in patients of the training cohort (n= 27). While presence of extrahepatic metastases shows a trend of impaired survival (85 vs 50 days, hazard ratio (HR): 0.453, 95% confidence interval (95% CI):.153- 1.33; p= .14), only detection of CTC was significantly associated with an impaired OS (168 vs. 39.5 days, HR:.38, 95% CI:.148-.99; p= .048).

Median follow-up was 85 days (range: 3- 981), three patients (12.5%) were alive at the end of follow-up at day 981. Median OS was significantly longer in CTC negative patients (168 vs. 39 days, hazard ratio.38, 95% confidence interval:.15-.99; p =.048). Kaplan-Meier curves estimating OS are shown in **Figure 2**.

In the RC, thirteen patients were enrolled undergoing curative-intended surgery between October 2017 and April 2018. Two blood samples for each patient were obtained prior to surgery: Blood samples were taken simultaneously by puncturing a peripheral vein (pv) and from a central venous (cv) catheter that had been placed at the day of scheduled surgery. Patients, in whom diagnosis of CCA or GBCA was not confirmed histologically (n = 2), were excluded from further analysis. Out of eleven patients who were included in final analysis, nine (82%) were male, median age was 67 years. The most common tumor type was pCCA (n = 6) and the ECOG status was 0 in nine patients (82%). CTC were found in six patients (55%). In two patients, CTCs were found in pv and cv compartment, while in two patients each, CTCs were only found in pv or the cv compartment. Taken together, by adding CTCs measurement in cv compartment, two patients had been additional identified to be CTC positive.

**Figure 3:**
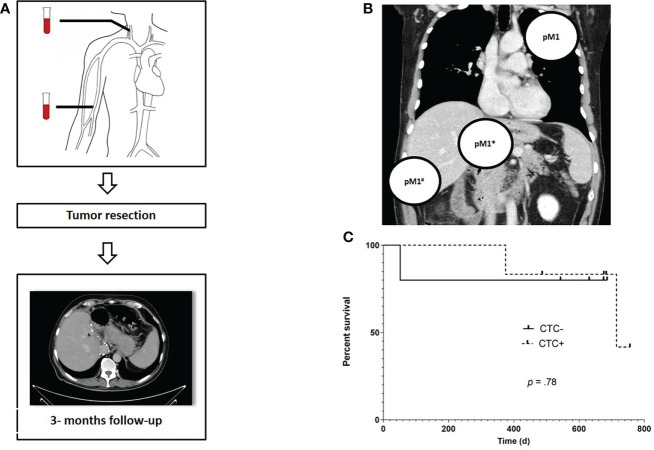
Study algorithm, localization of prior occult metastases and overall survival (OS) for FC patients. Prior to surgery, blood samples for CTC measurement were obtained from the central and peripheral venous compartment. Following tumor resection, a CT scan was scheduled every three months for routine followup **(A)**. Localization of metastases that were not detectable at the initial radiology staging but were found during or immediately after tumor resection, e.g. pulmonary filiae (pM1), a distant metastasis infiltrating the duodenum (pM1*) and local peritoneal carcinomatosis (pM1#) in a total of three CTC + patients **(B)**. Kaplan – Meier estimates reveals no significant difference in terms of OS between CTC positive and negative RC patients (HR: 0.74; 95% CI:.04- 12.28 **(C)**.

Patients were followed for a median time of 446 days (range: 43- 537 days). In three patients, distant metastases were found intraoperatively (n = 2) or soon after surgery (**Figure 3**). In all of these patients, CTCs were found prior to surgery while none of the CTC negative patients developed metastases within the follow-up period. Three patients (27%) died during follow up, two of these were initially diagnosed positive for CTCs and both were found to have an advanced, non-resectable tumor during exploration. The third patient died due to an unrelated cause. No significant difference was found regarding OS between CTC positive (CTC +) and negative (CTC -) patients in the RC with a median OS of 751 days in CTC + patients (mOS in CTC negative patients is not yet reached, p = 0.78; hazard ratio: 0.74; 95% confidence interval: 0.044-12). Characteristics of RC cohort are show in **Table 2**.

## Discussion

CCA is a rare, but aggressive primary liver malignancy, characterized by a poor prognosis and high recurrence rate (35). Specific biomarkers are therefore needed, not only for primary diagnosis but also to guide treatment, especially since the established tumor marker carbohydrate antigen 19-9 (CA 19-9) has a low diagnostic accuracy in early-stage cancer and several non-malignant conditions can also cause elevated CA 19-9 levels (36, 37).

In this study, we demonstrated that CTC are detectable in patients with cholangio- and gallbladder carcinoma and that evidence of CTC has a prognostic impact in patients with progressed tumor disease. Furthermore, we found that CTC detection in patients undergoing curative intended tumor resection is associated with occult tumor spreading and metastases.

Using a stepwise approach, we first measured CTC in a cohort of patients with metastatic tumor disease. In this cohort, we found CTC in 44% of all patients, underlining the feasibility of CTC detection using the CellSearch System. In comparison to previous studies by Yang et al. and Ustwani et al., reporting detection rates of 17% and 25%, respectively, the number of CTC positive patients in our study was relevant higher (30, 31). Addressing this, it has to be mentioned, that in both studies a threshold of ≥2 CTCs had been used to classify patients as CTC positive while we used a cut-off value of ≥ 1 CTC to diagnose patients positive. The rationale for using this threshold was based on recent studies including patients with hepatocellular- or urothelial carcinoma and the ABC-03 trial, investigating the impact of biomarkers in biliary tract cancer patients receiving cediranib, applying a cut-off value ≥ 1 CTC (28, 38, 39).

Furthermore, it has to be mentioned that the amount of detectable CTCs varies among different tumor types and also depends on tumor extend: while larger amounts with > 5 CTCs were found in patients with metastatic colorectal or breast cancer, Yang et al. found a median of one or more CTC in 25 out of 88 CCA patients (28%) (40, 41). According to this, in our study CTCs were more often detected in patients with an advanced tumor stage, while in the RC cohort, primarily one CTC per blood sample was detected in CTC positive patients.

As the prognostic relevance of CTC for CCA patients has been demonstrated in the study of Yang et al., the median OS of CTC positive patients with progressed CCA was also significantly reduced in our study (31). Hence, we can confirm the finding of Yang et al. in European cohort. Interestingly, other risk factors for poor prognosis in patients with metastatic tumor disease, such as distant metastasis or positive lymph node status, did not significantly affect the median OS in our study. But given the comparably small size of the training cohort and the inhomogeneity of the patient’s tumor stage, further studies including a greater number of patients are needed to further address this question.

After proofing the prognostic impact of CTCs in the first phase of the study, we evaluated the diagnostic relevance of CTC in patients with localized CCA presenting for curative intended tumor resection. Prior to surgery, an accurate tumor staging in CCA patients is important, because lymph node metastases are a significant negative predictor for postoperative OS (42). But as radiological imaging has a poor sensitivity to detect local lymph node infiltration (43), additional biomarkers are needed to improve preoperative assessment of tumor spread. The association of CTCs and micrometastases has been evaluated in different tumor types, but has not yet been evaluated in CCA and GBCA patients.

In the RC cohort, we found CTCs in >50% of all patients, but only one CTC positive patient was found to have positive lymph nodes (N1). Interestingly, only in CTC positive patients, local tumor progression (e.g., infiltrating of the duodenum in one patient, a local peritoneal carcinomatosis undetectable by CT scan prior to surgery in another patient) was diagnosed during surgery. Furthermore, in another CTC positive patient, distant pulmonary metastases were found within a few days following surgery, when an additional CT scan was performed to rule out pulmonary embolism. Thus, preoperative detection of CTC should raise suspicion of advanced tumor spread. In summary, while presence of CTCs does not seem to correlate with lymph node status, CTC positive patients seemed to be at risk for occult tumor progression, invisible on imaging techniques.

In all previous studies investigating CTCs in CCA patients, blood specimen for CTC measurement had only been obtained from peripheral cubital veins. But distribution of CTC varies among different vascular compartments in various tumor types: in patients with colorectal cancer who underwent tumor resection, a larger amount of CTCs had been detected in the mesenteric blood compared to the pv compartment (44). Furthermore, in a study of Fang et al. including HCC patients presenting for transarterial chemoembolization (TACE), CTCs were found significantly more often in the central venous than in the peripheral venous compartment (45). Considering this remarkably different distribution, we obtained blood samples from RC patients from a peripheral and a central vein in parallel prior to surgery.

In the RC, we found no difference in terms of the amount of CTCs detected in pv and cv compart-ment: only one CTC was found in each CTC positive sample. But it has to be noted that in two patients, CTCs were only detected in cv but not in pv blood samples, pointing to a possibly different distribution of CTCs in cv compartment. On the other hand, in two other CTC positive patients, cells had only been detected in pv compartment, whereas cv blood samples had been negative for CTCs. Taken together, this finding demonstrates, that sequential testing of patients increases the amount of CTC but studies with a larger number of patients are needed to further elucidate CTC concentration in different vascular compartments.

Regarding the relatively small number of CTCs that had been detected in patients undergoing curative resection, several aspects have to be taken into account: First, the amount of CTCs that are shed in the blood stream correlates with the tumor size (46). Regarding the often small size of BTC, this will limit the number of CTCs a priori. Second, measuring CTCs by using an EpCAM-based system can be ham-pered by the fact, that CTCs partially underwent a complex process, termed epithelial- to mesenchymal transition (EMT), in which CTCs are changing their epithelial properties to more stem cell or mesench-mal attributes, including downregulation of surface proteins such as EpCAM, and thus potentially limiting CTC detection (47–49).

This study has several limitations, that need to be addressed. First, the FC consisted of patients with different types of CCA in terms of their anatomical localization and also patients with GBCA. Given the fact that iCCA is associate with an increased risk of mortality compared to dCCA, distribution of different types of CCA might have interfered survival rates. Additionally, lymph node status and presence of distant metastases differed among patients in the trainings cohort. Second, FC patients were heterogeneous regarding the treatment modalities and this may also contribute to clinical course and outcome: while some of the patients were receiving palliative intended chemotherapy, others were only treated symptomatically without any anti-tumor directed medication. And although blood samples for CTC measurement were only taken shortly before chemotherapy was administered, one cannot rule out the impact of chemotherapy on CTC spread or shedding. Most notably, the FC and RC both consist of only a small number of patients, partially due to the relative rare tumor entity, hampering further statistical subgroup analysis.

Taken together and considering the limitations mentioned, in this pilot study using a two-step approach, we demonstrated that CTCs are detectable and associated with impaired OS in a Western cohort of patients with progressed and metastatic CCA. Furthermore, in a prospective second part of the study, preoperative detection of CTCs was associated with the presence of metastases that had been untraceable in previous imaging. Additionally, we demonstrated that CTCs are detectable in both, the central and the peripheral venous compartment.

Although preoperative detection of CTCs was not associated with an impaired OS in our study, the prognostic impact of this biomarker should be further evaluated in a large cohort of patients undergoing curative-intended resection.

## Data availability statement

The datasets presented in this study can be found in online repositories. The names of the repository/repositories and accession number(s) can be found below: https://osf.io/a3yt5/.

## Ethics statement

The studies involving human participants were reviewed and approved by Institutional Review Board of the Ethik-Kommission der Ärztekammer Hamburg, Hamburg, Germany (PV-3578). The patients/participants provided their written informed consent to participate in this study.

## Author contributions

Conceptualization, KS, TF and HW. Methodology, KS, JF and TF. Software, JF and TF. Formal analysis, KS, SR, TF, JF. Investigation, TF, JF, JK AH, JL and KS. Resources, KS and HW. Data curation TF, JF JK and HW. Writing—original draft preparation, TF, JF and KS. Writing—review and editing, JL, SH, AL, KP, SR and HW. Visualization, TF and JF. Supervision, KS, HW, SH and AL. Project administration, KS and HW. All authors contributed to the article and approved the submitted version.

## Acknowledgments

We like to thank Cornelia Coith for technical support and assistance, Ines Gil Ibanez and Felix Piecha for acquisition of data and Prof. Dr. M. Quante for critically revising the study draft.

## Conflict of interest

The authors declare that the research was conducted in the absence of any commercial or financial relationships that could be construed as a potential conflict of interest.

## Publisher’s note

All claims expressed in this article are solely those of the authors and do not necessarily represent those of their affiliated organizations, or those of the publisher, the editors and the reviewers. Any product that may be evaluated in this article, or claim that may be made by its manufacturer, is not guaranteed or endorsed by the publisher.

## References

[1] Banales JM Marin JJG Lamarca A Rodrigues PM Khan SA Roberts LR. Cholangiocarcinoma 2020: The next horizon in mechanisms and management. Nat Rev Gastroenterol Hepatol (2020) 17(9):557–88. doi: 10.1038/s41575-020-0310-z PMC7447603 32606456

[2] Shaib Y El-Serag HB. The epidemiology of cholangiocarcinoma. Semin Liver Dis Mai (2004) 24(2):115–25. doi: 10.1055/s-2004-828889 15192785

[3] Banales JM Cardinale V Carpino G Marzioni M Andersen JB Invernizzi P. Expert consensus doc-ument: Cholangiocarcinoma: current knowledge and future perspectives consensus statement from the European network for the study of cholangiocarcinoma (ENS-CCA). Nat Rev Gastroenterol Hepa-tol (2016) 13(5):261–80. doi: 10.1038/nrgastro.2016.51 27095655

[4] Khan AS Dageforde LA. Cholangiocarcinoma. Surg Clinics (2019) 99(2):315–35. doi: 10.1016/j.suc.2018.12.004 30846037

[5] DeOliveira ML Cunningham SC Cameron JL Kamangar F Winter JM Lillemoe KD. Cholangiocarci-noma: thirty-one-year experience with 564 patients at a single institution. Ann Surg (2007) 245(5):755–62. doi: 10.1097/01.sla.0000251366.62632.d3 PMC1877058 17457168

[6] Nakeeb A Pitt HA Sohn TA Coleman J Abrams RA Piantadosi S. Cholangiocarcinoma. a spec-trum of intrahepatic, perihilar, and distal tumors. Ann Surg (1996) 224(4):463–73; discussion 473-475. doi: 10.1097/00000658-199610000-00005 8857851 PMC1235406

[7] Bismuth H Nakache R Diamond T. Management strategies in resection for hilar cholangiocarcinoma. Ann Surg (1992) 215(1):31–8. doi: 10.1097/00000658-199201000-00005 PMC1242367 1309988

[8] Clements O Eliahoo J Kim JU Taylor-Robinson SD Khan SA. Risk factors for intrahepatic and extrahe-patic cholangiocarcinoma: A systematic review and meta-analysis. J Hepatol (2020) 72(1):95–103. doi: 10.1016/j.jhep.2019.09.007 31536748

[9] Petrick JL Yang B Altekruse SF Van Dyke AL Koshiol J Graubard BI. Risk factors for intrahepatic and extrahepatic cholangiocarcinoma in the united states: A population-based study in SEER-Medicare. PloS One (2017) 12(10):e0186643. doi: 10.1371/journal.pone.0186643 29049401 PMC5648218

[10] Shin HR Oh JK Masuyer E Curado MP Bouvard V Fang YY. Epidemiology of cholangiocarcinoma: an update focusing on risk factors. Cancer Sci (2010) 101(3):579–85. doi: 10.1111/j.1349-7006.2009.01458.x PMC11158235 20085587

[11] Bertuccio P Turati F Carioli G Rodriguez T La Vecchia C Malvezzi M. Global trends and predic-tions in hepatocellular carcinoma mortality. J Hepatol (2017) 67(2):302–9. doi: 10.1016/j.jhep.2017.03.011 28336466

[12] Yao KJ Jabbour S Parekh N Lin Y Moss RA. Increasing mortality in the united states from cholangio-carcinoma: an analysis of the national center for health statistics database. BMC Gastroenterol (2016) 16(1):117. doi: 10.1186/s12876-016-0527-z 27655244 PMC5031355

[13] Strijker M Belkouz A van der Geest LG van Gulik TM van Hooft JE de Meijer VE. Treatment and survival of resected and unresected distal cholangiocarcinoma: A nationwide study. Acta Oncol (2019) 58(7):1048–55. doi: 10.1080/0284186X.2019.1590634 30907207

[14] Ruys AT van Haelst S Busch OR Rauws EA Gouma DJ van Gulik TM. Long-term survival in hilar chol-angiocarcinoma also possible in unresectable patients. World J Surg (2012) 36(9):2179–86. doi: 10.1007/s00268-012-1638-5 PMC3414707 22569746

[15] Bird N Elmasry M Jones R Elniel M Kelly M Palmer D. Role of staging laparoscopy in the stratifi-cation of patients with perihilar cholangiocarcinoma. Br J Surg (2017) 104(4):418–25. doi: 10.1002/bjs.10399 27861766

[16] Coelen RJS Ruys AT Besselink MGH Busch ORC van Gulik TM. Diagnostic accuracy of staging laparos-copy for detecting metastasized or locally advanced perihilar cholangiocarcinoma: a systematic review and meta-analysis. Surg Endosc (2016) 30(10):4163–73. doi: 10.1007/s00464-016-4788-y PMC5009158 26895909

[17] Groot Koerkamp B Wiggers JK Gonen M Doussot A Allen PJ Besselink MGH. Survival after re-section of perihilar cholangiocarcinoma-development and external validation of a prognostic nomo-gram. Ann Oncol (2015) 26(9):1930–5. doi: 10.1093/annonc/mdv279 PMC4754626 26133967

[18] van Vugt JLA Gaspersz MP Coelen RJS Vugts J Labeur TA de Jonge J. The prognostic value of portal vein and hepatic artery involvement in patients with perihilar cholangiocarcinoma. HPB (Ox-ford) (2018) 20(1):83–92. doi: 10.1016/j.hpb.2017.08.025 28958483

[19] Liu J Lian J Chen Y Zhao X Du C Xu Y. Circulating tumor cells (CTCs): A unique model of cancer metastases and non-invasive biomarkers of therapeutic response. Front Genet (2021) 12:734595. doi: 10.3389/fgene.2021.734595 34512735 PMC8424190

[20] Heidrich I Abdalla TSA Reeh M Pantel K. Clinical applications of circulating tumor cells and circulat-ing tumor DNA as a liquid biopsy marker in colorectal cancer. Cancers (2021) 13(18):4500. doi: 10.3390/cancers13184500 34572727 PMC8469158

[21] Pantel K Alix-Panabières C. Liquid biopsy and minimal residual disease — latest advances and implica-tions for cure. Nat Rev Clin Oncol (2019) 16(7):409–24. doi: 10.1038/s41571-019-0187-3 30796368

[22] Raimondi C Nicolazzo C Gradilone A Giannini G De Falco E Chimenti I. Circulating tumor cells: Exploring intratumor heterogeneity of colorectal cancer. Cancer Biol Ther (2014) 15(5):496–503. doi: 10.4161/cbt.28020 24521660 PMC4026071

[23] Bork U Rahbari NN Schölch S Reissfelder C Kahlert C Büchler MW. Circulating tumour cells and outcome in non-metastatic colorectal cancer: a prospective study. Br J Cancer (2015) 112(8):1306–13. doi: 10.1038/bjc.2015.88 PMC4402459 25867263

[24] Lalmahomed ZS Mostert B Onstenk W Kraan J Ayez N Gratama JW. Prognostic value of circulat-ing tumour cells for early recurrence after resection of colorectal liver metastases. Br J Cancer (2015) 112(3):556–61. doi: 10.1038/bjc.2014.651 PMC4453661 25562435

[25] Dotan E Alpaugh RK Ruth K Negin BP Denlinger CS Hall MJ. Prognostic significance of MUC-1 in circulating tumor cells in patients with metastatic pancreatic adenocarcinoma. Pancreas (2016) 45(8):1131–5. doi: 10.1097/MPA.0000000000000619 PMC4983223 26967453

[26] Hou JM Krebs MG Lancashire L Sloane R Backen A Swain RK. Clinical significance and molecular characteristics of circulating tumor cells and circulating tumor microemboli in patients with small-cell lung cancer. J Clin Oncol (2012) 30(5):525–32. doi: 10.1200/JCO.2010.33.3716 22253462

[27] Cristofanilli M Budd GT Ellis MJ Stopeck A Matera J Miller MC. Circulating tumor cells, disease progression, and survival in metastatic breast cancer. N Engl J Med (2004) 351(8):781–91. doi: 10.1056/NEJMoa040766 15317891

[28] von Felden J Schulze K Krech T Ewald F Nashan B Pantel K. Circulating tumor cells as liquid bi-omarker for high HCC recurrence risk after curative liver resection. Oncotarget (2017) 8(52):89978–87. doi: 10.18632/oncotarget.21208 PMC5685725 29163804

[29] Schulze K Gasch C Staufer K Nashan B Lohse AW Pantel K. Presence of EpCAM-positive circulat-ing tumor cells as biomarker for systemic disease strongly correlates to survival in patients with hepa-tocellular carcinoma. Int J Cancer (2013) 133(9):2165–71. doi: 10.1002/ijc.28230 23616258

[30] Al Ustwani O Iancu D Yacoub R Iyer R. Detection of circulating tumor cells in cancers of biliary origin. J Gastrointest Oncol (2012) 3(2):97–104. doi: 10.3978/j.issn.2078-6891.2011.047 22811877 PMC3397649

[31] Yang JD Campion MB Liu MC Chaiteerakij R Giama NH Ahmed Mohammed H. Circulating tumor cells are associated with poor overall survival in patients with cholangiocarcinoma. Hepatology (2016) 63(1):148–58. doi: 10.1002/hep.27944 PMC4684812 26096702

[32] Osta WA Chen Y Mikhitarian K Mitas M Salem M Hannun YA. EpCAM is overexpressed in breast cancer and is a potential target for breast cancer gene therapy. Cancer Res (2004) 64(16):5818–24. doi: 10.1158/0008-5472.CAN-04-0754 15313925

[33] Went PT Lugli A Meier S Bundi M Mirlacher M Sauter G. Frequent EpCam protein expression in human carcinomas. Hum Pathol (2004) 35(1):122–8. doi: 10.1016/j.humpath.2003.08.026 14745734

[34] Kawashima R Abei M Fukuda K Nakamura K Murata T Wakayama M. EpCAM- and EGFR-targeted selective gene therapy for biliary cancers using Z33-fiber-modified adenovirus. Int J Cancer (2011) 129(5):1244–53. doi: 10.1002/ijc.25758 21710497

[35] Sirica AE Gores GJ Groopman JD Selaru FM Strazzabosco M Wei Wang X. Intrahepatic cholan-giocarcinoma: Continuing challenges and translational advances. Hepatology (2019) 69(4):1803–15. doi: 10.1002/hep.30289 PMC6433548 30251463

[36] Galli C Basso D Plebani M. CA 19-9: Handle with care. Clin Chem Lab Med (2013) 51(7):1369–83. doi: 10.1515/cclm-2012-0744/html 23370912

[37] Kim HS Han Y Kang JS Kang YH Lee M Sohn HJ. Serum carcinoembryonic antigen and carbohy-drate antigen 19-9 as preoperative diagnostic biomarkers of extrahepatic bile duct cancer. BJS Open (2021) 5(6):zrab127. doi: 10.1093/bjsopen/zrab127 34935900 PMC8693162

[38] Soave A Riethdorf S Dahlem R Minner S Weisbach L Engel O. Detection and oncological effect of circulating tumour cells in patients with variant urothelial carcinoma histology treated with radical cystectomy. BJU Int (2017) 119(6):854–61. doi: 10.1111/bju.13782 28182321

[39] Backen AC Lopes A Wasan H Palmer DH Duggan M Cunningham D. Circulating biomarkers dur-ing treatment in patients with advanced biliary tract cancer receiving cediranib in the UK ABC-03 trial. Br J Cancer (2018) 119(1):27–35. doi: 10.1038/s41416-018-0132-8 29925934 PMC6035166

[40] Gkountela S Szczerba B Donato C Aceto N. Recent advances in the biology of human circulating tu-mour cells and metastasis. ESMO Open (2016) 1(4):e000078. doi: 10.1136/esmoopen-2016-000078 27843628 PMC5070257

[41] Giuliano M Giordano A Jackson S Hess KR De Giorgi U Mego M. Circulating tumor cells as prognostic and predictive markers in metastatic breast cancer patients receiving first-line systemic treatment. Breast Cancer Res (2011) 13(3):R67. doi: 10.1186/bcr2907 21699723 PMC3218956

[42] Nishio H Nagino M Nimura Y. Surgical management of hilar cholangiocarcinoma: the Nagoya experi-ence. HPB (Oxford) (2005) 7(4):259–62. doi: 10.1080/13651820500373010 PMC2043097 18333203

[43] Noji T Kondo S Hirano S Tanaka E Suzuki O Shichinohe T. Computed tomography evaluation of re-gional lymph node metastases in patients with biliary cancer. Br J Surg (2008) 95(1):92–6. doi: 10.1002/bjs.5920 17853509

[44] Denève E Riethdorf S Ramos J Nocca D Coffy A Daurès JP. Capture of viable circulating tumor cells in the liver of colorectal cancer patients. Clin Chem (2013) 59(9):1384–92. doi: 10.1373/clinchem.2013.202846 23695297

[45] Fang ZT Zhang W Wang GZ Zhou B Yang GW Qu XD. Circulating tumor cells in the central and peripheral venous compartment &ndash; assessing hematogenous dissemination after transarterial chemoembolization of hepatocellular carcinoma. OncoTargets Ther (2014) 7:1311–8. doi: 10.2147/OTT.S62605 PMC4111660 25071374

[46] Chang YS di Tomaso E McDonald DM Jones R Jain RK Munn LL. Mosaic blood vessels in tumors: frequency of cancer cells in contact with flowing blood. Proc Natl Acad Sci USA (2000) 97(26):14608–13. doi: 10.1073/pnas.97.26.14608 PMC18966 11121063

[47] Kalluri R Weinberg RA. The basics of epithelial-mesenchymal transition. J Clin Invest (2009) 119(6):1420–8. doi: 10.1172/JCI39104 PMC2689101 19487818

[48] Mentis AFA Kararizou E. Metabolism and cancer: an up-to-date review of a mutual connection. Asian Pac J Cancer Prev (2010) 11(6):1437–44. 21338177

[49] Jie XX Zhang XY Xu CJ. Epithelial-to-mesenchymal transition, circulating tumor cells and cancer metastasis: Mechanisms and clinical applications. Oncotarget (2017) 8(46):81558–71. doi: 10.18632/oncotarget.18277 PMC5655309 29113414

